# Exploring the potential role of C‐peptide in type 2 diabetes management

**DOI:** 10.1111/dme.15469

**Published:** 2025-01-11

**Authors:** YeunYi Lin, Rory J. McCrimmon, Ewan R. Pearson

**Affiliations:** ^1^ School of Medicine University of Dundee, Ninewells Hospital & Medical School Dundee Scotland

**Keywords:** C‐peptide, type 2 diabetes mellitus, insulin secretion

## Abstract

Type 2 diabetes (T2D) is a complex condition characterised by the interaction between insulin resistance and beta cell dysfunction. C‐peptide, a key biomarker of endogenous insulin secretion, has a role in diagnosing type 1 diabetes (T1D). However, its utility in T2D has not been extensively studied. This review provides an overview of the progression of C‐peptide levels over time in T2D and discuss its interpretation in clinical settings. We reviewed current evidence on the relationship between C‐peptide levels and response to antidiabetic drugs, as well as the utility of C‐peptide testing in T2D treatment strategies. We also reviewed available evidence for C‐peptide in predicting future outcomes in T2D. In this review, we hoped to clarify the value of C‐peptide testing in understanding and managing T2D and to highlight areas where further research is needed.

**What's new?**

- Despite increasing availability of C‐peptide testing, its utility in the type 2 diabetes (T2D) population, particularly among non‐insulin‐treated individuals, remains underexplored
- While C‐peptide thresholds are well‐established for assessing insulin needs primarily in insulin‐treated individuals, their applicability in T2D and the clinical significance of higher levels beyond these thresholds are unclear
- The progression of C‐peptide levels over time in T2D demonstrates interindividual variability, likely reflecting disease heterogeneity and the influence of confounders such as insulin resistance
- More research is needed to evaluate the benefit of C‐peptide testing in predicting drug response and future insulin requirement

## 1 INTRODUCTION

C‐peptide is a short chain polypeptide secreted by pancreatic beta cells into the portal circulation in equimolar amount as insulin, following enzymatic cleavage of proinsulin. The measurement of peripheral C‐peptide has been widely accepted as a more reliable biomarker of beta cell function compared to peripheral insulin. This is because it has a longer plasma half‐life (7–8 times longer than plasma insulin), more stable plasma concentrations (contributed by constant peripheral clearance and negligible hepatic extraction) and its assays are not affected by exogenous insulin.^1^, ^2^, ^3^

The increasing availability and accessibility to C‐peptide immunoassay has led to a surge in the frequency of C‐peptide testing in clinical practice. This testing can be conducted using either blood or urine samples. Traditionally, stimulated C‐peptide was deemed to be the preferred method for assessment of beta cell function, with the mixed meal tolerance test currently recognised as the gold standard. However, alternative methods such as random C‐peptide (paired with glucose) or measuring C‐peptide levels after the largest home meal, which are strongly correlated with stimulated C‐peptide levels may be more practical.^4^, ^5^, ^6^, ^7^, ^8^, ^9^

In the era of precision medicine, there has been a recent emphasis on the role of C‐peptide in more accurately diagnosing diabetes type. For example, in a Scottish centre, C‐peptide testing led to the reclassification of diagnosis in 6.8% of individuals with type 1 diabetes (T1D) to other types of diabetes, such as type 2 diabetes (T2D) and monogenic diabetes.^10^ Importantly, this allowed optimisation of treatment resulting in better glycaemic control for these individuals.

Beyond its role in precision diagnosis to identify individuals with severe insulin deficiency, its clinical use in the care of people with T2D is less well‐defined. Therefore, although a recent comprehensive review on the utility of C‐peptide was performed by Maddaloni et al., we aimed to review the clinical utility of C‐peptide specifically in individuals diagnosed with T2D.^11^ We highlighted uncertainties surrounding the interpretation of C‐peptide results in T2D, and covered current evidence on aspects such as using C‐peptide to predict response to pharmacotherapies or to guide treatment strategies in T2D. We also highlighted some of the gaps in the literature in these aspects.

## 2 SEARCH METHODOLOGY

In this review, we included articles published up to September 2023 identified from PubMed using the following search terms (used alone or in combination): ‘C‐peptide’, ‘T2D’, ‘treatment response’, ‘treatment effect’, ‘glycaemic response’, ‘glycaemic control’, ‘glycaemic outcome’, ‘insulin management’, ‘insulin cessation’, ‘insulin withdrawal’, ‘insulin discontinuation’, ‘simplification’, ‘switch’, ‘glycaemic variability’, ‘hypoglycaemia’, ‘time below range’, ‘time in range’. Relevant articles cited by these articles were reviewed. Only articles published in English were included. During the revision process, an article from January 2024 was included as suggested by one of the reviewers.

## 3 C‐PEPTIDE VARIABILITY OVER TIME IN T2D

The onset of T2D has been suggested to result from declining insulin sensitivity followed by a gradual decrease in beta cell function eventually resulting in decompensated overt hyperglycaemic state.^12^, ^13^ After the diagnosis of T2D, estimates of beta cell function was found to decline over time in UKPDS, Belfast Diet Study and IRAS study populations when assessed with homeostatic model assessment (HOMA) modelling (using fasting insulin) and acute insulin response.^14^, ^15^, ^16^ However, when C‐peptide value is used independently as an indicator of beta cell function, without being included in model estimates, its association with the duration of T2D becomes less consistent. Whilst cross‐sectional studies showed that individuals with longer duration of T2D tend to exhibit lower levels of fasting C‐peptide, longitudinal studies had reported varied trends, with some showing stable C‐peptide levels over time (up to 20 years) and others indicating variable progression.^17^, ^18^, ^19^, ^20^, ^21^, ^22^, ^23^ For example, in one study approximately 50% of the participants had a reduction in their fasting C‐peptide, whereas the remaining 50% saw either an increase or no change.^22^ There is a possibility that individuals with significant decline in their C‐peptide levels may have a different underlying diabetes aetiology, but these studies were not designed specifically to assess the characteristics of this subgroup. Another potential explanation for the observed variability in C‐peptide levels over time is the differences in insulin sensitivity progression over time among individuals with T2D, as C‐peptide reflects both beta cell function and insulin sensitivity. For example, insulin sensitivity remained unchanged in the UKPDS and Belfast Diet Study but declined in the IRAS study.^14^, ^15^, ^16^ Additionally, the IMI‐DIRECT study found that participants with T2D became more insulin resistant over time.^24^

### 3.1 Summary

In summary, although beta cell function tends to decline over time following a diagnosis of T2D, C‐peptide levels do not necessarily reflect this progression. This may be partly because parallel changes in insulin sensitivity also affect C‐peptide concentrations (see below).

## 4 THE INTERPRETATION OF C‐PEPTIDE RESULTS IN T2D

C‐peptide levels increase from baseline (fasting state) in response to stimuli that augment endogenous insulin secretion, such as meals or specific drugs like arginine or glucagon. Maddaloni et al. have compared fasting, random and stimulated C‐peptide levels in their review, so this will not be discussed further here.^11^ A random C‐peptide measurement, which strongly correlates with stimulated C‐peptide (gold standard), is an appealing alternative method for testing in clinical practice.^5^, ^9^ Currently, there are no specific guideline recommendations for C‐peptide testing in adults with T2D. However, guidelines for T1D suggest using random samples (within 5 h of prior meal) with paired glucose, beyond 3 years from diagnosis, to assess severe insulin deficiency.^25^, ^26^, ^27^, ^28^ This testing is recommended when classification of diabetes remains uncertain, such as in adults suspected of having T1D or have rapid insulin requirement but with negative pancreatic autoantibodies. This is important because up to 35% of adults who develop T1D are initially misclassified as having T2D.^25^, ^26^, ^27^, ^28^

**FIGURE 1:**
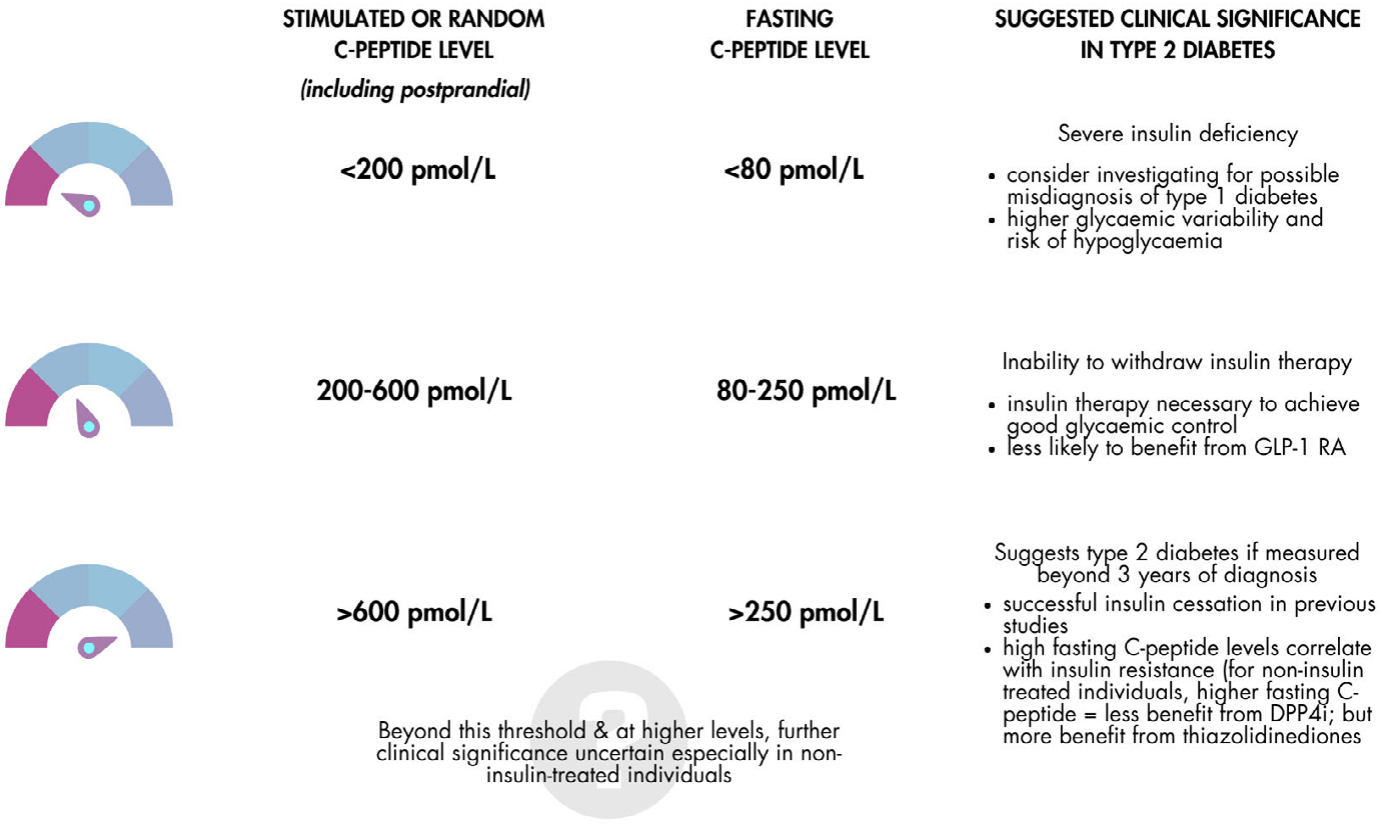
Random/stimulated and equivalent fasting C‐peptide levels and suggested clinical significance in T2D. GLP‐1 RA, glucagon‐like peptide‐1 receptor agonist; DPP4i, dipeptidyl peptidase‐4 inhibitor.

C‐peptide has been best validated in differentiating T1D or insulin‐requiring diabetes from non‐insulin requiring diabetes. Guidance on estimated cut‐off values are available in a review article by Jones et al. as well as guidelines for T1D, although both cautioned against over‐interpretation of values close to the suggested thresholds.^25^, ^29^ (see Figure 1) A non‐fasting serum C‐peptide <200 pmol/L (equivalent to fasting C‐peptide threshold <80 pmol/L) indicates near‐absolute insulin deficiency. Levels between 200 and 600 pmol/L (fasting threshold 80–250 pmol/L) are likely to correlate with T1D or monogenic diabetes but may also occur in insulin‐treated T2D. The upper limit of this range is arbitrary, estimated from previous studies on different populations. Non‐fasting (stimulated and random) serum C‐peptide values ranging from 500–970 pmol/L distinguished between insulin‐requiring and non‐insulin‐requiring diabetes.^4^, ^5^, ^10^, ^28^ In addition, it is recognised that there can be significant variability between laboratories in C‐peptide measurement; particularly at higher levels of C‐peptide. While the variations in C‐peptide results across most modern assays are generally modest, certain assays still produce substantially different results, with a maximum difference of 35% as shown in a recent comparison of five different assays.^30^ Hence, C‐peptide thresholds used at each centre may need to be adjusted depending on the assay used at the local laboratory. Efforts by various international organisations are currently underway to harmonise and standardise C‐peptide measurement.^31^ Despite the uncertainty around these thresholds, they have been successfully utilised in studies, for example, to clarify diabetes aetiology and in some cases, have allowed for successful insulin withdrawal.^10^, ^32^, ^33^

When interpreting C‐peptide results in T2D, it is essential to consider that C‐peptide is a composite measure of beta cell function and insulin resistance. Previous experimental studies identified that greater insulin resistance is associated with increased fasting insulin in a non‐linear relationship. Any minimal decrease in insulin sensitivity is associated with a large increase in fasting insulin level.^34^ Studies on individuals with T2D also identified correlations between fasting C‐peptide levels and markers of insulin resistance.^17^, ^35^ A similar association was also observed in a study population without a history of diabetes.^36^ Thus, fasting C‐peptide levels, like fasting insulin, tend to rise as insulin sensitivity decreases, making fasting C‐peptide more valuable for assessing insulin resistance.

Other than taking into account the underlying degree of insulin resistance, interpreting non‐fasting C‐peptide levels in T2D may also require considerations of other factors such as prior meals. Endogenous insulin secretion may also be influenced by concomitant oral antidiabetic medications, such as secretagogues, though guidelines have not recommended withholding these medications before C‐peptide testing. For example, a randomised, double‐blinded, crossover trial demonstrated that sulphonylureas elevated postprandial serum insulin and C‐peptide concentrations (up to two‐fold compared to placebo), with observed differences in concentration rise between glipizide and glibenclamide.^37^ GLP‐1 receptor agonists (GLP‐1 RA) principally act by augmenting insulin secretion and delaying gastric emptying, hence, theoretically may influence C‐peptide levels. However, data on this is lacking. A proof‐of‐concept study previously showed raised C‐peptide concentrations lasting several hours following the administration of inhaled GLP‐1 RA.^38^ Additionally, insulin may influence glycaemic control, subsequently reducing endogenous insulin secretion and C‐peptide levels. However, the impact of withholding rapid insulin on stimulated C‐peptide in insulin‐treated individuals is modest.^39^ Current guidelines do not recommend omitting insulin for the purpose of C‐peptide testing, especially given the potential implications of insulin omission.

**FIGURE 2:**
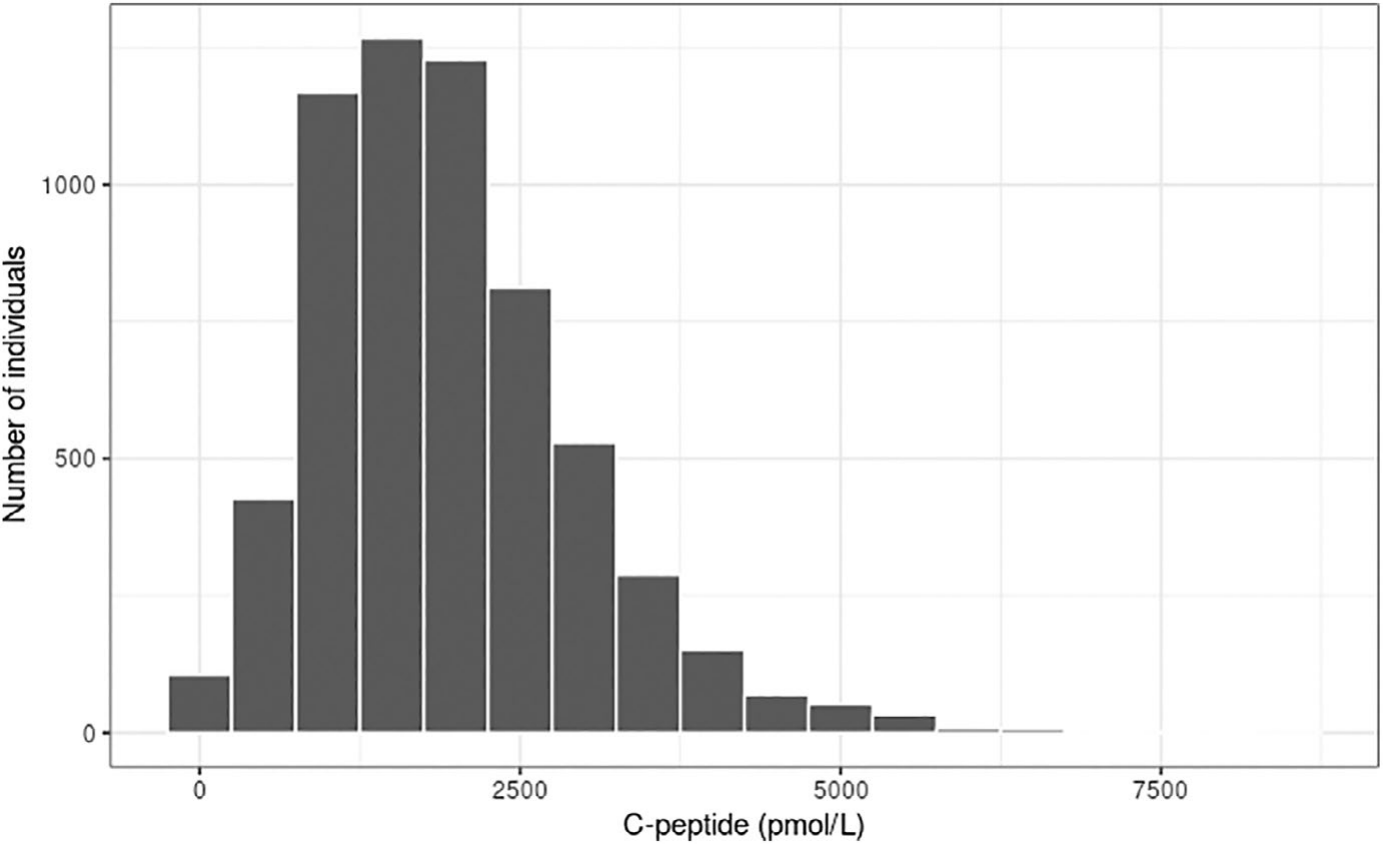
Random C‐peptide levels of 6150 individuals (mean age 67.0 years and mean diabetes duration 7.1 years) with clinically diagnosed T2D and estimated glomerular filtration rate ≥30 mL/min/1.73m^2^ in Tayside and Fife, Scotland.

While C‐peptide proves valuable in distinguishing between individuals requiring insulin therapy from those who do not, the significance of higher levels of C‐peptide levels, particularly when random C‐peptide is measured, remains unclear. As a reference, we included unpublished random C‐peptide concentrations in 6150 individuals with clinically diagnosed T2D from Tayside and Fife region, Scotland [see Figure 2]. There is a considerable variation in C‐peptide level in individuals with T2D particularly above the level of 1000 pmol/L and many people with T2D have very high C‐peptide concentrations, exceeding 1800 pmol/L. This emphasizes the heterogeneity of T2D. However, given that these are C‐peptide levels obtained in a non‐fasting state, it is important to consider the influence of other stimuli as previously highlighted.

The interpretation of C‐peptide level may be complicated by estimated glomerular filtration rate (eGFR) as C‐peptide is renally cleared.^11^, ^29^ During a study to evaluate the value of C‐peptide in the classification of diabetes in a cohort with end stage renal disease (ESRD) on haemodialysis, 84.6% (11 out of 13) of participants with a clinical classification of T1D were found to have C‐peptide level above 500 pmol/L. Participants who were clinically classified with T1D or T2D had similar mean C‐peptide levels (3080 +/− 1970 vs. 3370 +/− 1810 pmol/L respectively).^40^

### 4.1 Summary

In current clinical practice, C‐peptide testing is recommended for classifying diabetes diagnoses, with sufficient evidence supporting the established thresholds primarily in T1D or insulin‐treated individuals. However, some uncertainties remain regarding the interpretation of C‐peptide levels or application of these thresholds for individuals with T2D especially for those who are not treated with insulin. It is unclear to what extent adjustments should be made for underlying insulin resistance and concomitant medications. Additionally, beyond the lower thresholds used to categorize severe insulin deficiency and insulin requirement, the clinical significance of extremely high C‐peptide levels remains uncertain.

## 5 C‐PEPTIDE AND T2D SUBGROUPS

The identification of T2D clusters in the Swedish population highlighted the diverse nature of the disease and shifted the perspective away from classifying T2D as a homogeneous disease entity.^41^ Estimation of insulin resistance and beta cell function using HOMA modelling (based on fasting C‐peptide) were used to stratify participants in the study. Within the Swedish population, individuals in the mild‐obesity related diabetes (MOD) cluster demonstrated a gradual decline in fasting C‐peptide over time, in contrast to those in the mild age‐related diabetes (MARD) cluster which demonstrated minimal increase over time. This highlights that some of the heterogeneity observed in longitudinal C‐peptide levels may reflect subpopulations with varying natural histories.^41^ Subsequently, in IMI‐RHAPSODY, random C‐peptide and clinically ready markers such as HDL‐cholesterol, age at first visit, body mass index, and HbA1C were used as alternatives to HOMA assessments to reproduce the subgroups, with sensitivities ranging from 79.1% to 90.7% when compared to the Swedish clusters.^42^ Hence, by potentially assigning individuals to the subgroups with different metabolic profiles using C‐peptide and other clinically accessible markers, clinicians may provide a more personalised treatment strategy. Furthermore, the stratification provides insight into the disease progression and risks of complications for the subgroup. However, the clinical utility of subgroups allocation remains a matter of debate. The pathophysiology underlying T2D is a continuous process and each individual carries a unique genetic and environmental predisposition to diabetes. Models employing continuous simple and practical clinical measures (gender, BMI, age at diagnosis and HbA1c) have also demonstrated comparable or better predictions of diabetes outcomes or treatment responses compared to the cluster model.^43^

### 5.1 Summary

Considering C‐peptide alongside other clinical measures may enable for more targeted treatment and provide insight into disease progression for individuals with T2D through subgroup allocation. However, subgroup allocation does not necessarily outperform the use of simple and readily available continuous clinical phenotypes in predicting patient outcomes or drug treatment responses. The extent to which C‐peptide level enhances these existing models remains unclear.

## 6 UTILITY OF C‐PEPTIDE IN THE TREATMENT OF T2D

In this section, we aimed to consolidate the findings regarding the associations between C‐peptide and responses to non‐insulin or insulin therapy shedding light on the prospective use of C‐peptide in guiding management of T2D.

### 6.1 NON‐INSULIN THERAPY

Previous studies have attempted to evaluate treatment responses to non‐insulin therapy using C‐peptide, with a greater body of literature assessing responses to incretin‐based therapies such as glucagon‐like peptide‐1 receptor agonists (GLP‐1 RA) and dipeptidyl peptidase‐4 inhibitors (DPP4i). There was no article studying the treatment response to sodium‐glucose cotransporter‐2 inhibitors (SGLT2i) in T2D using C‐peptide.

### 6.2 Treatment response to GLP‐1 RA

C‐peptide has been recognised as a possible predictor of glycaemic response to GLP‐1 RA in multiple studies, primarily in insulin treated patients with severe insulin deficiency, rather than in non‐insulin treated patients. For instance, in a large prospective study on individuals with clinical diagnosis of T2D newly started on GLP‐1 RA, severe insulin deficiency (assessed using fasting C‐peptide) and presence of GAD/IA2 antibodies were associated with less HbA1C reduction in insulin‐treated individuals.^44^ Another smaller prospective study found that although postprandial UCPCR was not significantly correlated to HbA1C reduction in response to GLP‐1RA in univariate analysis, it became significantly associated after adjusting for baseline HbA1C. This result was found to be mainly driven by poorer glycaemic response among individuals with severe insulin deficiency when sensitivity analysis was performed. The authors highlighted that UCPCR may be a ‘rule out’ test for starting GLP‐1 RA in insulin treated individuals with severe insulin deficiency.^45^

A recent robust analysis performed by the GRADE (Glycaemic Reduction Approaches in Diabetes: A Comparative Effectiveness Study) study group helped address some of the gap in evidence on beta cell function and insulin sensitivity on the response to various therapies in non‐insulin‐treated individuals. The study group examined the impact of beta cell function (assessed with C‐peptide response during oral glucose tolerance test) and insulin sensitivity (HOMA2‐%S using fasting C‐peptide) on glycaemic control based on initial 3‐month HbA1C reduction and HbA1C progression rate over 5 years.^46^, ^47^ Individuals with T2D on metformin were randomised to insulin glargine, glimepiride, liraglutide or sitagliptin, but only the non‐insulin treatment groups were included in the analysis. This study concluded that both beta cell function and insulin sensitivity influence glycaemic response to these treatments. Specifically, in the liraglutide treatment arm, greater C‐peptide response during oral glucose tolerance test indicating better beta‐cell function was associated with better initial HbA1C reduction and slower long‐term HbA1C decline, with or without adjusting for insulin sensitivity.^46^

Instead of assessing beta cell function, Germain et al. used C‐peptide as a method to assess insulin resistance in non‐insulin treated cohort by measuring the time needed to reduce serum C‐peptide level by 50% during an insulin tolerance test. They found that insulin resistance is associated with less HbA1C change with liraglutide.^48^ However, insulin tolerance test may not be clinically practical. When evaluation was performed on clinical characteristics associated with insulin resistance (such as BMI, triglycerides, HDL‐cholesterol, sex hormone binding globulin and HOMA2%S) in non‐insulin‐treated individuals by the PRIBA (Predicting Response to Incretin Based Agents) study group, no significant association was identified between these factors and HbA1C change from GLP‐1 RA.^44^, ^49^ In the GRADE study, although HOMA2‐S% showed some influence on short‐term glycaemic control, it did not have a lasting impact on HbA1C progression.^46^

### 6.3 Treatment response to DPP4i

The studies included in this section on treatment response to DPP4i focuses on non‐insulin‐treated population, as the majority of the available evidence in literature pertains to this population.

Dennis et al. demonstrated that higher fasting C‐peptide level and post meal UCPCR were associated with less HbA1C reduction from DPP4i treatment (1.67 mmol/mol [0.2%] decrease in HbA1C response at 6 months per SD higher baseline fasting C‐peptide; 1.65 mmol/mol [0.2%] decrease in HbA1C at 6 months response per SD higher UCPCR) but there was no association with HOMA2‐B%. On the other hand, HOMA2‐IR and other markers of insulin resistance (such as BMI, triglycerides, and lower sex‐hormone binding globulin level) were associated with poorer glycaemic response to DPP4i.^49^ The effect of insulin resistance was also replicated in the GRADE analysis study. However, while better C‐peptide response in the GRADE study demonstrated a higher initial HbA1C reduction, it did not influence long‐term HbA1C progression.^46^ Thus, insulin sensitivity seems to have a greater impact on the treatment response to DPP4i compared to beta cell function in predominantly non‐Asian populations.^50^, ^51^ However, studies conducted on Asian population, which typically had lower baseline BMI (mean BMI 24.1–25.2 kg/m^2^), did not share consistent findings on this aspect. While some studies showed a better glycaemic response with lower BMI, no relationship was found with other markers of insulin resistance (including fasting C‐peptide) or HOMA‐IR.^50^, ^51^, ^52^

### 6.4 Treatment response to thiazolidinediones

There is consistent evidence suggesting that individuals with higher insulin resistance benefit more from thiazolidinediones.^53^, ^54^, ^55^ Specifically, higher fasting C‐peptide was associated with better responses to pioglitazone in a randomised controlled trial where participants were randomised to pioglitazone or acarbose. Fasting C‐peptide, fasting glucose, HbA1C and BMI were identified as independent predictors of response in multivariate logistic regression analysis.^56^

### 6.5 Treatment response to metformin

The evidence on the impact of beta cell function on glycaemic response to metformin is weak and shows contradicting results, with study designs varying markedly among the limited number of studies. In a randomised study comparing pioglitazone and metformin as second‐line treatments in addition to sulphonylurea, participants experienced greater HbA1C reduction when beta cell response, assessed using HOMA‐B, was reduced.^53^ In contrast, another randomised study observed that individuals with positive stimulated C‐peptide had a better response to the combination of metformin and sulphonylurea.^57^ Furthermore, multivariate regression analysis performed in another study did not include C‐peptide level as a significant factor for HbA1C reduction when diet‐controlled individuals are treated with a combination of metformin and sitagliptin.^58^

### 6.6 Treatment response to sulphonylurea

Better beta cell response is associated with better treatment outcomes with sulphonylurea.^46^, ^57^ In the GRADE study, a higher C‐peptide response to glucose was linked to greater initial HbA1C reduction as well as slower HbA1C progression.^46^

### 6.7 SGLT2i

In theory, C‐peptide may help identify insulin‐treated individuals with severe insulin deficiency or those misclassified as T2D, thus preventing the complication of euglycaemic ketoacidosis due to SGLT2i. However, there is currently no supporting evidence for this and guidelines have not recommended assessing endogenous insulin secretion before starting SGLT2i.^59^

## 7 INSULIN THERAPY

### 7.1 Treatment response to basal insulin

In a large post‐hoc analysis of 16 randomised controlled trials evaluating response to the addition of insulin glargine to suboptimally controlled insulin‐naïve individuals, fasting C‐peptide did not predict the achievement of target glycaemic control. Levels were similar in groups who achieved target glycaemic control and those who did not (mean fasting C‐peptide 1160 pmol/L in responders and 1140 pmol/L in non‐responders).^60^ The high levels of fasting C‐peptide in both groups could be due to hyperglycaemia during fasting state (mean glucose 10.8 mmol/L) and that participants recruited for this in this analysis were very insulin‐resistant.

### 7.2 Treatment escalation with insulin therapy

In current clinical practice, glycaemic control is typically intensified using a stepwise approach. The selection of second or subsequent line of treatment for achievement of glycaemic control is tailored to management goals, such as choosing higher efficacy drugs to achieve glycaemic goal, managing weight or avoiding hypoglycaemia. A potential alternative management pathway may involve stratifying patients to identify who would benefit most from insulin sensitisers or from the introduction or intensification of insulin therapy. For example, in a cross‐sectional study, individuals already treated with basal insulin responded better to prandial insulin if they had a lower stimulated C‐peptide level.^61^ Studies thus far had mainly shown an association between lower C‐peptide levels (fasting or non‐fasting) and insulin requirements in newly diagnosed individuals or those needing a more intensive regime.^62^, ^63^ However, the evidence on whether C‐peptide predicts future insulin requirement is limited. One study used various assessments of beta cell function that utilise C‐peptide to develop prediction models for achieving HbA1C target (<48 mmol/mol) with metformin monotherapy or need for insulin therapy. The model based on mean ratio of C‐peptide at 120 minutes to fasting C‐peptide (C‐peptide_120_/C‐peptide_0_) had the best model performance (AUC = 0.724 for metformin monotherapy; AUC = 0.776 for insulin requirement).^64^ However, it only slightly outperformed the baseline model that used variables such as age, gender, BMI and baseline HbA1C (AUC = 0.661 for metformin monotherapy, AUC = 0.677 for insulin requirement) and was also not adjusted for fasting glucose which was significantly different among treatment groups.

### 7.3 Insulin simplification or cessation

C‐peptide may be used to guide decisions on insulin cessation in clinical settings. A stimulated C‐peptide level of 600 pmol/L and fasting level of 300 pmol/L were used to guide successful insulin withdrawal in individuals with T2D with HbA1C up to 64 mmol/mol (positive predictive values of 76% and 90% respectively). Those with results below these thresholds are unlikely to be able to discontinue insulin.^4^, ^32^

For older adults, who are more vulnerable to treatment‐associated complications such as hypoglycaemia, treatment simplification is highly relevant to prevent such complications. Two intervention studies on older adults treated with insulin therapy used serum C‐peptide as a criterion for de‐escalation of treatment.^33^, ^65^ In a prospective cohort study, elderly individuals (mean age 80 +/− 6 years) with any recorded C‐peptide level above 360 pmol/L were recruited for insulin simplification where oral agents were added. In this study, 19 out of 35 participants were able to discontinue insulin completely while the number of insulin injections were reduced from an average of 2.7 +/− 1 injections/day to 1.5 +/− 0.8 in the remaining participants.^33^ Similarly, elderly participants (mean age 76 +/− 6 years) in another cohort study with positive stimulated C‐peptide and recorded hypoglycaemia on continuous glucose monitoring underwent simplification of their insulin regime with the substitution of prandial insulin using other non‐insulin therapy.^65^ In both studies, all participants transitioned off insulin treatment without clinically significant deterioration in HbA1C (mean baseline HbA1C levels between 60–65 mmol/mol) and reported a significant reduction in hypoglycaemia after insulin simplification. Improvement in HbA1C was observed in individuals with higher baseline HbA1C (above 64 mmol/mol [8.0%]) in both studies.^33^, ^65^

### 7.4 Risk of hypoglycaemia and glycaemic variability

Studies on the association between C‐peptide and glycaemic variability as well as hypoglycaemic risk provided further insights into management of people with insulin‐treated T2D. Multiple post‐hoc analyses were carried out to assess the outcomes of prescribing insulin glargine (100 U/mL or 300 U/mL) for insulin‐naive individuals who were unable to achieve target glycaemic control with oral antidiabetic medications, stratified according to baseline fasting C‐peptide.^66^, ^67^, ^68^, ^69^ People with higher fasting C‐peptide levels (more insulin‐resistant) were less prone to both overall and nocturnal hypoglycaemia (three times lower events per person‐year when C‐peptide was >1200 pmol/L compared to ≤400 pmol/L).^68^ This may manifest as early as the first 8 weeks after initiating insulin glargine.^66^ The negative correlation between fasting C‐peptide and hypoglycaemia event rate remains when individuals on concomitant sulphonylurea were excluded.^67^ Participants with lower fasting C‐peptide in the VADT trial were also more prone to variations in fasting glucose levels and more likely to report severe hypoglycaemia. Notably, they were also more likely to require insulin therapy for diabetes control, placing them at an increased risk of hypoglycaemia.^70^ Additional studies on individuals with insulin‐treated T2D using continuous glucose monitoring also demonstrated an inverse correlation between C‐peptide levels and coefficient of variation of glucose.^71^, ^72^, ^73^

In addition to findings above, data on C‐peptide and glycaemic variability in non‐insulin‐treated population is limited but some studies have explored the relationship between beta cell function and glycaemic variability. Lower endogenous insulin secretion was associated with higher plasma glucose during oral glucose tolerance test.^74^ Beta cell function did not impact on the glycaemic variability in those who are diet‐controlled when assessed using continuous glucose monitoring whereas in individuals treated with metformin and/or sulphonylurea, postprandial C‐peptide (adjusted for paired glucose level) contributes to glycaemic variability in addition to the type of treatment regime.^75^ However, as model performances were not reported, it is unclear whether postprandial beta cell function provides additional benefit in identifying glycaemic variation beyond clinical information on the treatment regime.

### 7.5 Summary

There is more evidence for using C‐peptide to assess treatment response with GLP‐1 RA and DPP4i, but very limited evidence for its use with metformin or SGLT2i.

Insulin‐treated individuals with severe insulin deficiency are unlikely to experience significant glycaemic benefits from GLP‐1 RA. Non‐insulin‐treated individuals, who are less likely to have severe insulin deficiency, benefit more from GLP‐1 RA if they show higher C‐peptide response to glucose stimulation. However, even those with less C‐peptide response may still gain benefits, such as weight reduction, from this treatment. Therefore, C‐peptide may be most useful to target treatment selection in insulin‐treated individuals when deciding to start GLP‐1 RA.

In predominantly non‐Asian population, individuals with higher fasting C‐peptide levels, indicative of greater insulin resistance, derive less benefit from DPP4i. This primarily applies to non‐insulin‐treated individuals. Studies on insulin‐treated individuals are lacking, perhaps because the use of DPP4i is less common in insulin‐treated individuals.

There is strong evidence that individuals who are insulin‐resistant benefit more from thiazolidinediones whereas sulphonylureas are likely to elicit a better response if there is still endogenous insulin secretion, likely related to its mechanism of action.

Currently, C‐peptide has not been established as a predictor of future insulin requirement. It may be used to guide decisions about whether individuals on basal insulin would benefit more from adding prandial insulin or other options such as insulin sensitisers. C‐peptide has been successfully used in small prospective studies to withdraw insulin, which will benefit frail or elderly individuals the most. In insulin‐treated individuals, C‐peptide may help identify those more likely to experience hypoglycaemia or higher glycaemic fluctuations. However, there is lack of comprehensive studies directly assessing the independent role of C‐peptide level as a marker for glycaemic variability in the non‐insulin‐treated population.

## 8 CONCLUSION

T2D is a complex and heterogeneous disease, as reflected by the variation in C‐peptide levels observed both at a single time point or over time. As C‐peptide has primarily been studied in individuals receiving insulin therapy, current established C‐peptide thresholds used in clinical practice are predominantly intended for this group. There is promising potential for C‐peptide in guiding management decisions, such as insulin cessation, but further exploration is needed in non‐insulin‐treated population. More concrete evidence is required to demonstrate how C‐peptide, alongside readily accessible clinical data, can predict patient outcomes, drug treatment responses and future insulin needs.

## CONFLICT OF INTEREST STATEMENT

YYL has no competing interest. RJM has received honoraria or speaking from Sanofi and Novo Nordisk. ERP has received honoraria for speaking from Novo Nordisk, Lilly and Illumina.

## Supporting information


Data S1.
