# Supplementary material for: Exploring the potential role of C‐peptide in type 2 diabetes management
Source: Diabet Med. 2025 Jan 11;42(3):e15469. doi: 10.1111/dme.15469 (PMC11823364; doi:10.1111/dme.15469)
Supplement: Supplementary file 1 — Data S1. [file DME-42-e15469-s001.docx]

**REFERENCES**

51. Kim SA, Shim WH, Lee EH, et al. Predictive clinical parameters for the therapeutic efficacy of sitagliptin in korean type 2 diabetes mellitus. *Diabetes &amp; metabolism journal*. 2011/04// 2011;35(2):159-165. doi:10.4093/dmj.2011.35.2.159

52. Bando Y, Kanehara H, Aoki K, Hisada A, Toya D, Tanaka N. Obesity may attenuate the HbA1c-lowering effect of sitagliptin in Japanese type 2 diabetic patients. *J Diabetes Investig*. Mar 28 2012;3(2):170-4. doi:10.1111/j.2040-1124.2011.00156.x

53. Nagasaka S, Aiso Y, Yoshizawa K, Ishibashi S. Comparison of pioglitazone and metformin efficacy using homeostasis model assessment. *Diabet Med*. Feb 2004;21(2):136-41. doi:10.1111/j.1464-5491.2004.01083.x

54. Jones TA, Sautter M, Van Gaal LF, Jones NP. Addition of rosiglitazone to metformin is most effective in obese, insulin-resistant patients with type 2 diabetes. *Diabetes, Obesity and Metabolism*. 2003;5(3):163-170. doi:<https://doi.org/10.1046/j.1463-1326.2003.00258.x>

55. Kim YM, Cha BS, Kim DJ, et al. Predictive clinical parameters for therapeutic efficacy of rosiglitazone in Korean type 2 diabetes mellitus. *Diabetes Res Clin Pract*. Jan 2005;67(1):43-52. doi:10.1016/j.diabres.2004.05.001

56. Blüher M, Lübben G, Paschke R. Analysis of the Relationship Between the Pro12Ala Variant in the PPAR-γ2 Gene and the Response Rate to Therapy With Pioglitazone in Patients With Type 2 Diabetes. *Diabetes Care*. 2003;26(3):825-831. doi:10.2337/diacare.26.3.825

57. Hermann LS, Scherstén B, Melander A. Antihyperglycaemic efficacy, response prediction and dose-response relations of treatment with metformin and sulphonylurea, alone and in primary combination. *Diabet Med*. Dec 1994;11(10):953-60. doi:10.1111/j.1464-5491.1994.tb00253.x

58. Lim S, An JH, Shin H, et al. Factors predicting therapeutic efficacy of combination treatment with sitagliptin and metformin in type 2 diabetic patients: the COSMETIC study. *Clin Endocrinol (Oxf)*. Aug 2012;77(2):215-23. doi:10.1111/j.1365-2265.2011.04240.x

59. Committee ADAPP. 9. Pharmacologic Approaches to Glycemic Treatment: Standards of Care in Diabetes—2024. *Diabetes Care*. 2023;47(Supplement_1):S158-S178. doi:10.2337/dc24-S009

60. Owens DR, Landgraf W, Frier BM, et al. Commencing insulin glargine 100 U/mL therapy in individuals with type 2 diabetes: Determinants of achievement of HbA1c goal less than 7.0%. *Diabetes, Obesity and Metabolism*. 2019;21(2):321-329. doi:<https://doi.org/10.1111/dom.13607>

61. Jones A. Assessment of endogenous insulin secretion in insulin treated diabetes predicts postprandial glucose and treatment response to prandial insulin. *BMC Endocrine Disorders*. 2012;12(6)

62. Dario T, Riccardo G, Silvia P, et al. The utility of assessing C-peptide in patients with insulin-treated type 2 diabetes: a cross-sectional study. *Acta Diabetologica*. 2021/04/01 2021;58(4):411-417. doi:10.1007/s00592-020-01634-1

63. Bell DSH, Ovalle F. Outcomes of initiation of therapy with once-daily combination of a thiazolidinedione and a biguanide at an early stage of type 2 diabetes. *Diabetes, Obesity and Metabolism*. 2004;6(5):363-366. doi:<https://doi.org/10.1111/j.1462-8902.2004.00357.x>

64. Abdelgani S, Puckett C, Adams J, Triplitt C, DeFronzo RA, Abdul-Ghani M. Insulin Secretion Predicts the Response to Antidiabetic Therapy in Patients With New-onset Diabetes. *J Clin Endocrinol Metab*. Nov 19 2021;106(12):3497-3504. doi:10.1210/clinem/dgab403

65. Munshi MN, Slyne C, Segal AR, Saul N, Lyons C, Weinger K. Simplification of Insulin Regimen in Older Adults and Risk of Hypoglycemia. *JAMA Internal Medicine*. 2016;176(7):1023. doi:10.1001/jamainternmed.2016.2288

66. Frier BM, Landgraf W, Zhang M, Bolli GB, Owens DR. Hypoglycaemia risk in the first 8 weeks of titration with insulin glargine 100 U/mL in previously insulin-naive individuals with type 2 diabetes mellitus. *Diabetes, Obesity and Metabolism*. 2018;20(12):2894-2898. doi:<https://doi.org/10.1111/dom.13450>

67. Bolli GB, Landgraf W, Bosnyak Z, Melas-Melt L, Home PD. Hypoglycaemia risk with insulin glargine 300 U/mL compared with glargine 100 U/mL across different baseline fasting C-peptide levels in insulin-naïve people with type 2 diabetes: A post hoc analysis of the EDITION 3 trial. *Diabetes, Obesity and Metabolism*. 2020;22(9):1664-1669. doi:<https://doi.org/10.1111/dom.14065>

68. Landgraf W, Owens DR, Frier BM, Zhang M, Bolli GB. Fasting C-peptide, a biomarker for hypoglycaemia risk in insulin-naïve people with type 2 diabetes initiating basal insulin glargine 100 U/mL. *Diabetes, Obesity and Metabolism*. 2020;22(3):315-323. doi:<https://doi.org/10.1111/dom.13897>

69. Landgraf W, Bigot G, Frier BM, Bolli GB, Owens DR. Response to insulin glargine 100 U/mL treatment in newly-defined subgroups of type 2 diabetes: Post hoc pooled analysis of insulin-naïve participants from nine randomised clinical trials. *Primary Care Diabetes*. 2023/08/01/ 2023;17(4):379-385. doi:<https://doi.org/10.1016/j.pcd.2023.04.010>

70. Koska J, Nuyujukian DS, Bahn GD, Zhou JJ, Reaven PD. Association of low fasting C-peptide levels with cardiovascular risk, visit-to-visit glucose variation and severe hypoglycemia in the Veterans Affairs Diabetes Trial (VADT). *Cardiovascular Diabetology*. 2021/12/08 2021;20(1):232. doi:10.1186/s12933-021-01418-z

71. Si Y, Shen Y, Lu J, et al. Impact of acute-phase insulin secretion on glycemic variability in insulin-treated patients with type 2 diabetes. *Endocrine*. 2020/04/01 2020;68(1):116-123. doi:10.1007/s12020-020-02201-y

72. Christensen MB, Gæde P, Hommel E, Gotfredsen A, Nørgaard K. Glycaemic variability and hypoglycaemia are associated with C-peptide levels in insulin-treated type 2 diabetes. *Diabetes & Metabolism*. 2020/02/01/ 2020;46(1):61-65. doi:<https://doi.org/10.1016/j.diabet.2019.02.002>

73. Hope SV, Knight BA, Shields BM, et al. Random non-fasting C-peptide testing can identify patients with insulin-treated type 2 diabetes at high risk of hypoglycaemia. *Diabetologia*. Jan 2018;61(1):66-74. doi:10.1007/s00125-017-4449-2

74. Ferrannini E, Gastaldelli A, Miyazaki Y, Matsuda M, Mari A, DeFronzo RA. β-Cell Function in Subjects Spanning the Range from Normal Glucose Tolerance to Overt Diabetes: A New Analysis. *The Journal of Clinical Endocrinology & Metabolism*. 2005;90(1):493-500. doi:10.1210/jc.2004-1133

75. Kohnert KD, Augstein P, Zander E, et al. Glycemic variability correlates strongly with postprandial beta-cell dysfunction in a segment of type 2 diabetic patients using oral hypoglycemic agents. *Diabetes Care*. Jun 2009;32(6):1058-62. doi:10.2337/dc08-1956
